# Water Cannot Activate Traps of the Carnivorous Sundew Plant *Drosera capensis*: On the Trail of Darwin’s 150-Years-Old Mystery

**DOI:** 10.3390/plants12091820

**Published:** 2023-04-28

**Authors:** Andrej Pavlovič, Ondřej Vrobel, Petr Tarkowski

**Affiliations:** 1 Department of Biophysics, Faculty of Science, Palacký University, Šlechtitelů 27, CZ-783 71 Olomouc, Czech Republic; 2 Czech Advanced Technology and Research Institute, Palacký University, Šlechtitelů 27, CZ-783 71 Olomouc, Czech Republic; ondrej.vrobel@upol.cz (O.V.); petr.tarkowski@upol.cz (P.T.); 3 Centre of the Region Haná for Biotechnological and Agricultural Research, Department of Genetic Resources for Vegetables, Medicinal and Special Plants, Crop Research Institute, Šlechtitelů 29, CZ-783 71 Olomouc, Czech Republic

**Keywords:** abscisic acid, aspartic protease, carnivorous plant, digestive enzyme, jasmonic acid, sundew

## Abstract

In his famous book *Insectivorous plants*, Charles Darwin observed that the bending response of tentacles in the carnivorous sundew plant *Drosera rotundifolia* was not triggered by a drop of water, but rather the application of many dissolved chemicals or mechanical stimulation. In this study, we tried to reveal this 150-years-old mystery using methods not available in his time. We measured electrical signals, phytohormone tissue level, enzyme activities and an abundance of digestive enzyme aspartic protease droserasin in response to different stimuli (water drop, ammonia, mechanostimulation, chitin, insect prey) in Cape sundew (*Drosera capensis*). Drops of water induced the lowest number of action potentials (APs) in the tentacle head, and accumulation of jasmonates in the trap was not significantly different from control plants. On the other hand, all other stimuli significantly increased jasmonate accumulation; the highest was found after the application of insect prey. Drops of water also did not induce proteolytic activity and an abundance of aspartic protease droserasin in contrast to other stimuli. We found that the tentacles of sundew plants are not responsive to water drops due to an inactive jasmonic acid signalling pathway, important for the induction of significant digestive enzyme activities.

## 1. Introduction

Carnivorous plants of the genus sundew (*Drosera* sp.) capture insect prey by using sticky tentacles. The tentacles exhibit remarkably complex behaviour when they capture and digest insect prey. The heads of the marginal tentacles rapidly bend inward after mechanical stimulation. Rapid movement of the tentacles serves to hold captured insects on the leaf and is mediated by action potentials (APs) which are initiated by a receptor potential [1,2,3,4,5]. The APs are initiated just below the swollen head of the tentacle and propagated to its base, but not to the trap tissue [1,2,3,5]. A series of several APs are necessary to initiate an inflection of the tentacle [2]. Inflection brings the prey to the centre of the leaf and in contact with the short inner tentacles. The contact with the inner tentacles triggers the oscillation of membrane potentials and localized accumulation of jasmonates in the trap tissue [5,6]. Recent study revealed that this oscillation probably represents Ca^2+^ waves from individual tentacles [7], by means of which the individual tentacles communicate with each other. This results in the bending of all marginal tentacles that have no previous contact with the prey, thus safely fixing the prey [5,6]. The plant phytohormones from the group of jasmonates play an indispensable role in the bending reaction and initiation of digestive enzyme secretion [5,6,8]. The secretome of the carnivorous sundew plant consists of cysteine and aspartic proteases, chitinase, ribonuclease, phosphatase, alpha-galactosidase and β-1,3-glucanase [5,9,10,11,12,13].

The peculiarity of the insect-trapping behaviour of sundew plants attracted the attention of many early biologists. The first detailed experimental investigations on the common sundew (*Drosera rotundifolia*) were conducted by Charles Darwin and were described in his book *Insectivorous plants* [14]. Darwin showed that the tentacles are sensitive to both chemical and mechanical stimulation. He applied more than one hundred different organic and inorganic chemicals on the leaf and carefully observed the bending reactions of the sticky tentacles. He tested various nitrogenous and non-nitrogenous compounds in solution, and he found that the plant detects with almost unerring certainty the presence of nitrogen. In relation to these experiments, it was necessary first to ascertain the effect of distilled water, and he found that the more sensitive leaves are affected by it, but only slightly in comparison to nitrogenous compounds. Thus, the glands are insensible to the weight and repeated blows of drops of heavy rain, and the plants are thus likewise saved from much useless movement. On the other hand, the pressure exerted by the lighter human hair is sufficient for tentacle bending reactions. Darwin wrote about these observations: “*It would obviously have been a great evil to the plant if the tentacles were excited to bend by every shower of rain; but this evil has been avoided by the glands either having become through habit insensible to the blows and prolonged pressure of drops of water, or to their having been originally rendered sensitive solely to the contact of solid bodies*” [14].

Now, 150 years later, we can investigate the tentacle behaviour via more sophisticated methods in comparison to just observing its inflection. Recent studies have shown that the amount and type of enzymes secreted in sundew plants is regulated by different stimuli [5,9,15]. Already Darwin noticed that secretion induced by nitrogenous chemicals, but not mechanical stimuli, had the power to digest proteins, and that the glands of *Drosera* secrete: “*some ferment analogous to pepsin*” [14]. Recently, Darwin’s assumption was confirmed, and the enzyme in digestive fluid related to pepsin was discovered—aspartic protease droserasin [5,12,16]. We also know that even mechanical stimulation of tentacles and subsequent generation of APs is essential for the initial release of low amounts of digestive enzymes; however, their production is boosted after chemical sensing. Chitin and proteins are sensed the best and trigger the expression of digestive enzymes [5,9,15]. Here, we investigated the effect of water drops on the secretion of digestive enzymes in Cape sundew (*Drosera capensis*) to complement Darwin’s assertion of the inefficiency of water to induce the tentacle bending reaction. In addition, we also applied mechanical stimuli, ammonium chloride, chitin and live prey for comparison.

## 2. Results

### 2.1. Electrical Signals

**Figure 1:**
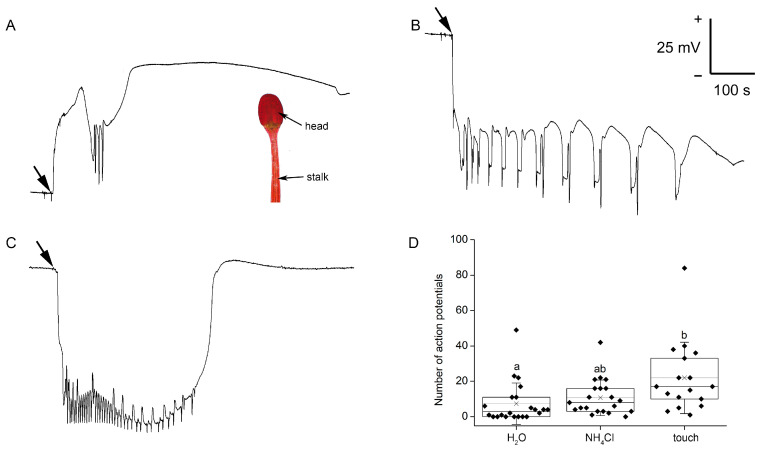
Extracellular recording of membrane potential from the tentacle head of the carnivorous sundew plant (*Drosera capensis*). (**A**) response to water drop application, with inset showing tentacle head and stalk; (**B**) response to drop of 50 mM of NH_4_Cl; (**C**) response to touch; and (**D**) box plots of the number of action potentials triggered by different stimuli recorded during a 700-s timespan. Boxplots show the individual measurements (diamonds), 25th percentile, median and 75th percentile of the data points. Thin vertical lines with crosses represent the means. Whiskers indicate ± 1 S.D. Different letters indicate significant differences at *p* < 0.05 (ANOVA, Tukey’s test), *n* = 17–22.

Application of a drop of distilled water on the marginal tentacle head resulted in membrane hyperpolarization (positive voltage shift recorded extracellularly, representing intracellular hyperpolarization). In many cases, a receptor potential with few APs (7.4 ± 11.7, mean ± S.D., min = 0, max = 49, *n* = 22) was also triggered (Figure 1A). On the other hand, application of 50 mM of NH_4_Cl resulted in the depolarization of membrane potentials (negative voltage shift recorded extracellularly, representing intracellular depolarization) which trigger the series of APs (10.7 ± 10.0, mean ± S.D., min = 0, max = 42, *n* = 21, Figure 1B). Touch or contact with any solid bodies induced the depolarization of a membrane potential (negative voltage shift recorded extracellularly, representing intracellular depolarization), probably a receptor potential (RP). The RP was accompanied by a series of APs (21.9 ± 20.2, mean ± S.D., min = 1, max = 87, *n* = 17). After that, the membrane potential repolarized (Figure 1C). The number of APs triggered by all these stimuli was highly variable (Figure 1D) and can be explained by the different sensitivities of the individual tentacles. Measurements of electrical signals in response to insect application was not measured due to the interference with the locomotion of the live insects.

### 2.2. Accumulation of Phytohormones

**Figure 2:**
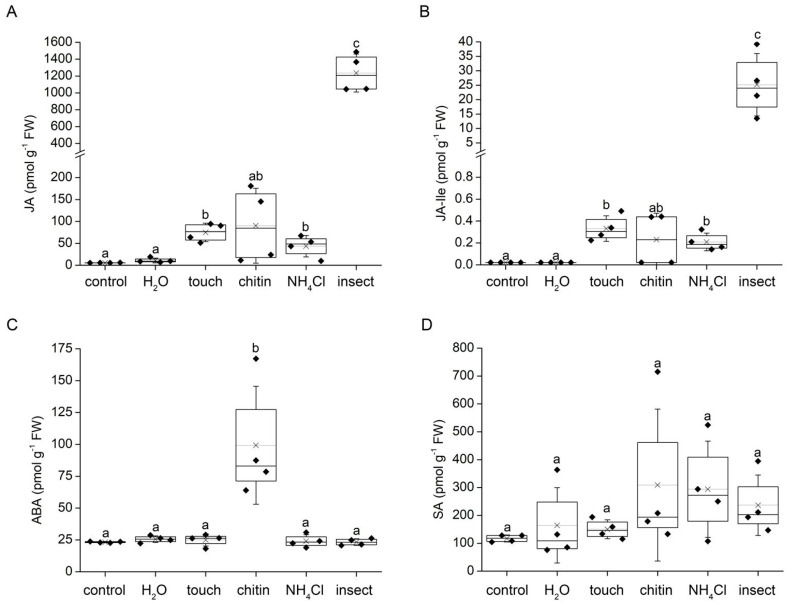
Trap tissue phytohormone accumulation in response to different stimuli in the carnivorous sundew plant (*Drosera capensis*) after 2 h. (**A**) jasmonic acid, (**B**) jasmonoyl-L-isoleucine, (**C**) abscisic acid, (**D**) salicylic acid. Boxplots show the individual measurements (diamonds), 25th percentile, median, and 75th percentile of the data points. Thin vertical lines with crosses represent the means. Whiskers indicate ± 1 S.D. Different letters indicate significant differences at *p* < 0.05 (ANOVA, Tukey’s test or if non-homogeneity was present using multiple comparison via Welch’s test), *n* = 4.

Drops of distilled water increased the average accumulation of jasmonic acid (JA) two-fold in comparison to control plants; however, the increase was not significant. Jasmonoyl-L-isoleucine (JA-Ile) remained below the limit of detection (LOD), similar to the control plants (<0.022 pmol g^−^^1^ FW). In contrast, the application of 50 mM of NH_4_Cl in the same drop of solution induced a significant, eight times higher accumulation of JA and at least a ten-fold increase in JA-Ile (calculated from LOD). Mechanical stimulation induced a 13-fold significant increase in JA and at least a 15-fold increase in JA-Ile. The flakes of chitin induced a similar response, a 16-fold higher accumulation of JA and at least a 10-fold increase in JA-Ile; however, due to the high variability of the samples, the increase was not significantly different in comparison to the control. A huge boost of JA and JA-Ile were detected after the application of live insect prey: a 220-fold and at least a 1100-fold increase, respectively (Figure 2A,B). The accumulation of abscisic acid (ABA) was increased fourfold only in response to chitin application (Figure 2C). The concentration of salicylic acid (SA) was not significantly affected at all (Figure 2D).

**Figure 3:**
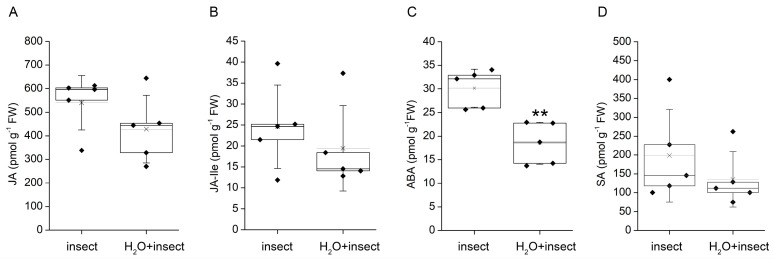
Trap tissue phytohormone accumulation in response to pre-treatment with water drops in the carnivorous sundew plant (*Drosera capensis*) after 2 h. The sundew traps had been pre-treated with three water drops 5 min before insect prey was applied (H_2_O + insect) and compared with traps fed on insect prey (insect). (**A**) jasmonic acid, (**B**) jasmonoyl-L-isoleucine, (**C**) abscisic acid, (**D**) salicylic acid. Boxplots show the individual measurements (diamonds), 25th percentile, median, and 75th percentile of the data points. Thin vertical lines with crosses represent the means. Whiskers indicate ± 1 S.D. Asterisks denote significant differences at *p* < 0.01 (**), Student’s *t*-test, *n* = 5.

We were interested in whether the drop of water inducing membrane hyperpolarization could bring the tentacles into the state of insensibility as suggested by Darwin [14]. When the drop of water had been applied 5 min before the live insect prey, we did not observe any differences in the tentacle and trap bending reaction in comparison to traps with applied live prey without water. There were not any significant changes in jasmonate accumulation after 2 h; only the content of ABA significantly decreased in water drop pre-treated plants, probably due to better water status (Figure 3).

### 2.3. Enzyme Activity

**Figure 4:**
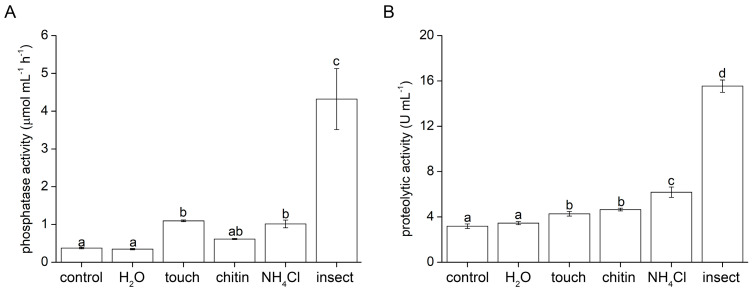
Enzyme activities in digestive fluids in response to different stimuli applied on the trap of the carnivorous sundew plant (*Drosera capensis*) after 24 h. (**A**) phosphatase activity, (**B**) proteolytic activity. Different letters denote significant differences at *p* < 0.05 (ANOVA, Tukey’s test), *n* = 4.

Phosphatase activity was not significantly increased in response to water drop application on the trap surface. However, the same drop of water containing 50 mM of NH_4_Cl significantly increased phosphatase activities. Mechanostimulation also increased phosphatase activities, but the flakes of chitin did not cause any additional effect. The best activator of phosphatase activities was the living insect prey (Figure 4A).

Proteolytic activity was also not significantly increased in response to water drop application on the trap surface (Figure 4B). All other stimuli significantly increased proteolytic activity and insect prey was the most effective one. The stimulatory effect of chitin was comparable with mechanical stimulation, indicating no additional chitin specific response on proteolytic activities.

### 2.4. Abundance of Digestive Enzyme

**Figure 5:**
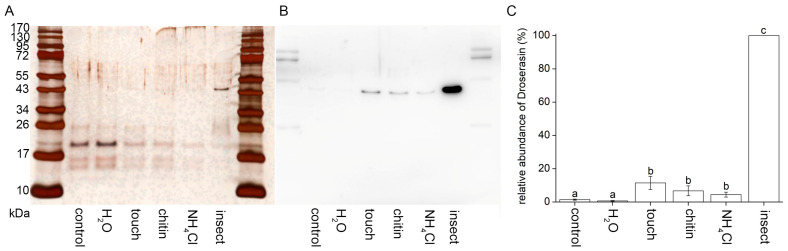
Immunodetection of aspartic protease (droserasin) in digestive fluid in response to different stimuli applied on the trap of the carnivorous sundew plant (*Drosera capensis*) after 24 h. The proteins were separated in 10% (*v*/*v*) sodium dodecyl sulphate–polyacrylamide gel electrophoresis (SDS-PAGE) and subjected to Western blot analysis. The same volume of digestive fluid was loaded (20 µL). (**A**) Silver-stained SDS-PAGE of digestive fluid in response to different stimuli. (**B**) Western blot analysis using antibodies against droserasin in response to different stimuli. (**C**) Quantification of chemiluminescence signal intensity. The results shown are representative of three independent experiments. Different letters denote significant differences at *p* < 0.05 (ANOVA, Tukey’s test), *n* = 3.

Aspartic and cysteine proteases are responsible for proteolytic activities in digestive fluid in sundew plants [5,12]. We successfully immunodetected aspartic protease droserasin. The overall trend in its accumulation in digestive fluid is in accordance with proteolytic activity measurements. The signal intensities were comparable between the control and water drop treated plants. Application of NH_4_Cl increased its abundance and mechanostimulation and chitin application had comparable signal intensities. Live insect prey induced the highest abundance of droserasin in digestive fluid (Figure 5).

## 3. Discussion

Charles Darwin in his famous book *Insectivorous plants* was the first to realize that the tentacles of sundew plants responded to water only very slightly, if at all [14]. We found that a drop of water induced the lowest number of APs in the tentacle. Because the tentacle bending reaction is proportional to the number of APs triggered [2], the weak reaction observed by Darwin could be caused by the low number of APs triggered. In contrast to the Venus flytrap, where a single touch induced a single AP [17], the sundew plant generates a series of APs in response to a single touch or any other stimuli [1,2,5]. None or a low number of APs triggered can be caused by a preceding rapid and long-lasting membrane hyperpolarization. This finding is consistent with the observations of Williams and Pickard [2], who observed the same response and found out that the probability of AP generation is decreased with membrane hyperpolarization and vice versa. Moreover, the number of APs generated by water drop application in our study can be overestimated due to the mechanical contact with the measuring electrode. In contrast to water, Charles Darwin found that the salts of ammonia are a powerful inducer of the tentacle bending reaction [14]. Application of NH_4_Cl induced rapid long-lasting membrane depolarization and triggered more APs in accordance with Williams and Pickard’s observations [2], which may also explain Darwin’s observations. Mechanical stimulation of tentacles with solid bodies induced a strong and rapid bending response, short-lasting membrane depolarization called RP and the highest number of APs (Figure 1).

Membrane depolarization and generation of electrical signals in plants is related to the accumulation of a plant defence hormone from the group of jasmonates [18]. In the carnivorous plants, the jasmonate signalling pathway was co-opted for the regulation of carnivorous response, e.g., expression and secretion of digestive enzymes [19,20,21,22]. Water application did not induce a significant accumulation of jasmonates (JA, JA-Ile, Figure 2), probably due to water-induced membrane hyperpolarization. However, this hyperpolarization had no significant effect on jasmonate accumulation after subsequent prey capture (Figure 3), in contrast to Darwin’s observation that a drop of water may bring the tentacles to a state of decreased responsiveness to mechanical stimuli [14]. Despite the fact that the weight of the applied water drop was ten-fold higher than polystyrene balls (treatment touch), the polystyrene balls induced a seven-fold and sixteen-fold higher accumulation of JA and JA-Ile, respectively (Figure 2). This is in line with Darwin’s observations that tiny and much lighter human hair can induce the tentacle bending reaction in contrast to a water drop [14]. All other stimuli also induced a several-fold increase in jasmonates. On the other hand, only chitin induced a significant accumulation of ABA in accordance with our recent study on the Venus flytrap [23].

In our previous study we found that the digestive enzyme aspartic protease droserasin is under the control of jasmonates [5], and we used it as a proxy for the quantification of the physiological response. The accumulation of droserasin in tentacle droplets is not enhanced by water application, but all other stimuli induced its accumulation. The living prey triggered the highest accumulation of droserasin and corresponding proteolytic activity. Previous studies on different carnivorous plant genera showed that the living insect prey is indeed the best inducer of enzymatic activities [5,24,25]. This is probably caused by the combination of mechanical and chemical stimulations delivered to digestive organs of carnivorous plants. Chitin, a typical component of insect exoskeletons, did not trigger any additional accumulation of droserasin and proteolytic activity, in comparison to mechanical stimulation (Figure 5). This weak response of chitin on the secretion of proteolytic enzymes was also documented previously in *Drosera rotundifolia*, *Dionaea muscipula*, *Nepenthes alata* and *Nepenthes* x *Mixta* [9,23,24,25]. On the other hand, the presence of protein in insect bodies is a much better inducer of proteolytic activities [23,25].

Although the application of water did not induce the digestive ability of the carnivorous sundew, it was recently documented that water spray applied to *Arabidopsis thaliana* plants is sufficient to activate gene expression changes through JA signalling [26]. In a subsequent study it was demonstrated that the trichomes are true mechanosensors and that a rain drop initiated Ca^2+^ waves concentrically propagated away from trichomes, triggering a downstream sequence of responses. However, the accumulation of JA and JA-Ile was rather low, and only 11.8% of raindrop-induced genes were JA-responsive [27]. This is in accordance with our study, where a water drop did not induce significant accumulation of jasmonates (Figure 2) and thus enzymatic activities, which are under their control and therefore were not upregulated (Figure 4). This indicates that weak activation of the JA signalling pathway by a water drop was already pre-established in non-carnivorous plants and co-opted during the evolution of jasmonate signalling by carnivorous plants [28]. Matsumura et al. [27] suggested that rain drop mechanostimulation activates JA signalling only partially, and that mechanosensitive genes are regulated rather by calmodulin-binding transcription activator (CAMTA) directly through an increased intracellular Ca^2+^ concentration [Ca^2+^]_cyt_. Procko and his group [7] recently succeeded in the transformation of *Drosera spathulata* with Ca^2+^ sensor GCaMP3, observing that the Ca^2+^ wave propagated away from the tentacles in response to insect prey, mechanical stimulation and also in response to a water drop; however, a direct comparison of signal intensity in response to different treatments is not available (Procko, pers. comm.). Thus, only the increase in [Ca^2+^]_cyt_ is probably not sufficient for the induction of high jasmonate accumulation and enzyme synthesis in carnivorous plants, and the combination with another cellular factor is also important.

## 4. Materials and Methods

### 4.1. Plant Material, Culture Conditions and Experimental Setup

The carnivorous plant *Drosera capensis* L. (Cape sundew) is a small, erect perennial sundew native to the Cape region of South Africa. Experimental plants were grown from seeds and cultivated in a growth chamber at the Department of Biophysics in Olomouc (Czech Republic). Well-drained peat moss in plastic pots, placed in a tray filled with distilled water to a depth of 1–2 cm, was used. Daily temperatures fluctuated in the range of 23–26 °C, the relative air humidity was between 60–80% and there was a light/dark regime (12 h of light at 100-μmol photons m^−^^2^ s^−^^1^ per 12 h of dark). The traps were treated with 3 drops of distilled water or a 50-mM solution of NH_4_Cl. The mechanical stimulation was performed by placing 3 small polystyrene balls on the trap surface. Three flakes of chitin from shrimp shells (95% acetylated, Sigma Aldrich, St. Louis, MO, USA) were placed in the same manner on the trap surface. The last treatment was feeding the plants with three living fruit flies (*Drosophila melanogaster*). They were cultured from eggs in a carbohydrate-rich medium (1 L composed of 87 g of corn meal, 15 g of agar, 25 g of dry yeast, 50 g of crystal sugar, 40 mL desinfection solution) at 23 °C, and before application, they were paralyzed with a cold treatment (4 °C for 10 min) for better manipulation. In an additional experiment, three drops of water had been applied 5 min before application of the fruit flies.

### 4.2. Extracellular Recording of Electrical Signals from Tentacle Head

The electrical signals were measured on the tentacle head using nonpolarizable Ag–AgCl surface electrodes (Scanlab Systems, Prague, Czech Republic) using a non-invasive device inside a Faraday cage as described previously in [5,29]. First, we established good electrical contact between the electrode and the head of a marginal tentacle. Sometimes, the mechanical contact with the electrode triggered a depolarization and series of APs, and in such a case, we had to wait for the restoration of the membrane potential. The tentacle head region was stimulated by a drop of distilled water, 50 mM of NH_4_Cl or a gentle mechanical touch simulating a contact with solid bodies. The reference electrodes were submerged in a dish filled with 1–2 cm of water beneath the pot. The electrodes were connected to channels of an amplifier that had been made in-house (gain: 1–1000, noise: 2–3 mV, bandwidth (−3 dB): 10^5^ Hz, response time: 10 µs, input impedance: 10^12^ Ω). The signals from the amplifier were transferred to an analogue-digital PC data converter (12-bit converter, ±10 V, PCA-7228AL supplied by TEDIA, Plzeň, Czech Republic), and the data were collected every 30 ms. The sensitivity of the device was 13 µV.

### 4.3. Quantitative Analysis of Phytohormones

Endogenous levels of phytohormones were quantified using the isotope dilution LC- MS/MS method [30] 2 h after treatments. The collected plant tissues were frozen in liquid nitrogen and ground using a mortar and pestle. The homogenized material was extracted and purified as described in [31]. Briefly, 25 mg of sample were extracted with 1 mL of ice cold 50% acetonitrile (ACN) containing a mixture of stable isotope-labelled standards. Unlabelled (SA, JA-Ile, ABA) and labelled standards (D4-SA, D2-JA-Ile, D6-ABA) were purchased from OlChemIm (Olomouc, Czech Republic); JA and D5-JA were purchased from Merck (Darmstadt, Germany). The extraction was performed with assistance of an ice-cold ultrasonic bath for 30 min. After centrifugation (20,000× *g*, 15 min, 4° C) samples were purified using SPE. Waters (USA) Oasis™ HLB columns (30 mg, 1 mL cartridge) were activated by 1 mL of MeOH and equilibrated by 1 mL of H_2_O and 1 mL of 50% ACN. During sample loading, the flow-through fraction was collected and pooled with the fraction from a single-washing step of 1 mL 30% ACN. Collected fractions were evaporated under a vacuum. If necessary, the dried samples were stored at −20 °C prior to analysis. For analysis, samples were resuspended in 40 µL of mobile phase, filtered through 0.2-µm microspins (Ciro, Deerfield Beach, FL, USA) and analyzed via LC-(+)ESI-MS/MS in the multiple reaction monitoring (MRM) mode. LC-MS/MS analysis was performed using a Nexera X2 modular liquid chromatograph system coupled to an MS 8050 triple quadrupole mass spectrometer (Shimadzu, Kyoto, Japan) via an electrospray interface. Chromatographic separation was performed using a reversed-phase analytical column, Waters CSH™ C18, 2.1 mm × 150 mm, 1.7 µm. The aqueous solvent A consisted of 15 mM of formic acid adjusted to a pH of 3.0 with ammonium hydroxide. Solvent B was pure ACN. Separation was achieved with gradient elution at a flow rate of 0.4 mL min^−1^ at 40 °C. Then, 0–1 min 20% B and 1–11 min 80% B linear gradients followed by washing and equilibration to initial conditions for a further 7 min were applied. If possible, up to 3 MRM transitions (1 quantitative, the others qualitative) were monitored for each analyte to ensure as much confidence as possible in the correct identification of analytes in the different plant matrices. Raw data were processed via Shimadzu software LabSolutions ver. 5.97 SP1. All data were log transformed to calculate the results. To reduce experimental biases, procedures included a randomized sample list, and the blinding was imposed on the analyst (OV).

### 4.4. Measurements of Enzyme Activities

After 24 h, the digestive fluid was collected from 8 leaves by submerging the part of leaves covered by tentacles into 1.5 mL of a 50-mM Na-acetate buffer (pH 5.0). To measure the activity of acid phosphatases, we used the chromogenic substrate 4-nitrophenyl phosphate (Sigma-Aldrich, St. Louis, MO, USA). The substrate was prepared in a 50-mM acetate buffer (pH 5.0), and the concentration was 5 mM. Then, 50 µL of the collected fluid was added to 500 μL of a 50-mM Na-acetate buffer (pH 5.0) and mixed with 400 μL of the substrate. As a control, 400 μL of the substrate solution was mixed with 550 μL of the Na-acetate buffer. Mixed samples were incubated at 25 °C for 1 h and then 160 μL of 1.0 N NaOH was added to terminate the reaction. Absorbance was measured at 405 nm with a Specord 250 Plus double-beam spectrophotometer (Analytik Jena, Germany). A calibration curve was determined using 4-nitrophenol.

The proteolytic activity of the leaf exudates was determined by incubating 150 μL of a sample with 150 μL of 2% (*w*/*v*) bovine serum albumin (BSA) in 200 mM of glycine-HCl (pH 3.0) at 37 °C for 1 h. The reaction was stopped by the addition of 450 µL of 5% (*w*/*v*) trichloroacetic acid (TCA). Samples were incubated on ice for 10 min and centrifuged at 20,000× *g* for 10 min at 4 °C. The amount of released non-TCA-precipitable peptides was used as a measure of proteolytic activity, which was determined by comparing the absorbance of the supernatant at 280 nm to a blank sample with a Specord 250 Plus double-beam spectrophotometer (Analytik Jena, Jena, Germany). One unit of proteolytic activity was defined as an increase of 0.001 per min in the absorbance at 280 nm [9].

### 4.5. SDS-PAGE and Western Blots

To detect and quantify aspartic protease (droserasin), a polyclonal antibody against this protein was raised in rabbits by Genscript (Piscataway, NJ, USA). The following amino acid sequence (epitope) based on data from mass spectrometry [5] was synthesized: (NH_2_-) SAIMDTGSDLIWTQC (-CONH_2_) by Genscript, Piscataway, NJ, USA. The sequence was coupled to a carrier protein (keyhole limpet haemocyanin, KLH) and injected into two rabbits each. The terminal cysteine of the peptide was used for conjugation. The rabbit serum was analyzed for the presence of antigen-specific antibodies using an ELISA test.

The digestive fluid collected for the enzyme assays was subjected to Western blotting. The samples were heated and denatured for 30 min at 70 °C and mixed with a modified Laemmli sample buffer to a final concentration of 50 mM of Tris-HCl (pH 6.8), 2% SDS, 10% glycerol, 1% β-mercaptoethanol, 12.5 mM of EDTA, and 0.02% bromophenol blue. The same volume of digestive fluid was electrophoresed in 10% (*v*/*v*) SDS polyacrylamide gel [32]. The proteins in the gels were either visualized via silver staining (ProteoSilver; Sigma Aldrich) or transferred from the gel to a nitrocellulose membrane (Bio-Rad, Hercules, CA, USA) using a Trans-Blot SD Semi-Dry Electrophoretic Transfer Cell (Bio-Rad). After blocking in TBS-T containing 5% BSA overnight, the membranes were incubated with the primary antibody for 1 h at room temperature, and after washing, the membrane was incubated with the secondary antibody: the goat antirabbit IgG (H + L)-horseradish peroxidase conjugate (Bio-Rad). Blots were visualized and chemiluminescence was quantified using an Amersham Imager 600 gel scanner (GE HealthCare Life Sciences, Tokyo, Japan).

### 4.6. Statistical Analyses

Before statistical analyses, the data were tested for homogeneity of variance (Brown-Forsythe test). If the homogeneity was fulfilled, Student’s *t*-test and one-way analysis of variance (ANOVA) with Tukey’s post hoc test was used (Origin 8.5.1, Northampton, MA, USA). If homogeneity was not present multiple comparisons via Welch’s test were used (Microsoft Excel).

## 5. Conclusions

In conclusion, water drops cannot activate the digestive process in the carnivorous sundew plant *D. capensis*. The reason is a subthreshold accumulation of jasmonates which control the expression of digestive enzymes. This is in line with evidence from experiments on non-carnivorous plants [27].

## Data Availability

Upon request, the data will be provided by the corresponding author.
